# Editorial: Interactions of the nervous system with bacteria, volume II

**DOI:** 10.3389/fnins.2023.1125176

**Published:** 2023-01-23

**Authors:** Elisa L. Hill-Yardin, Andreas M. Grabrucker, Ashley E. Franks

**Affiliations:** ^1^School of Health and Biomedical Sciences, STEM College, RMIT University, Bundoora, VIC, Australia; ^2^Department of Anatomy and Physiology, The University of Melbourne, Parkville, VIC, Australia; ^3^Department of Biological Sciences, University of Limerick, Limerick, Ireland; ^4^Health Research Institute, University of Limerick, Limerick, Ireland; ^5^Bernal Institute, University of Limerick, Limerick, Ireland; ^6^School of Life Sciences, La Trobe University, Bundoora, VIC, Australia

**Keywords:** nervous system, microbiome, autism (ASD), schizophrenia, neurodegenerative diseases, preclinical models, clinical research

## Introduction

Increasingly, the interface of the nervous system and bacteria is being investigated with respect to wellbeing, given that the communications between the gut and the brain are recognized to be integral in health and disease. In this topic, the microbial profiles were studied in relation to behavioral changes, genetic and environmental influences, as well as patient pathophysiology. The focus of these studies included Autism Spectrum Disorder (ASD), schizophrenia, and neurodegenerative diseases such as Alzheimer's Disease, Parkinson's Disease, Amyotrophic lateral sclerosis (ALS), and frontotemporal dementia (FTD).

Altered gut-brain signaling has been implicated in several brain disorders, such as autism spectrum disorders (ASD). Accordingly, gut microbiota are increasingly considered a promising therapeutic target in ASD. Liu et al. showed that GW4064, an agonist of the Farnesoid X receptor (FXR), ameliorates the ASD-like behavior of BTBR mice, an animal model for ASD. In particular, the administration of GW4064 positively affected social behaviors as well as increased microbial abundance in the feces. The high Firmicutes to Bacteroidetes ratio that has been proposed as a typical ASD phenotype was counteracted. In addition, GW4064 normalized elevated Lactobacillus and decreased Allobaculum levels. These data provide further evidence that targeting microbiota composition is a potential strategy for ameliorating ASD symptoms.

Schizophrenia (SZ) still has a largely unknown etiology and pathogenesis, but the gut microbiome is suggested to play a role. Ling et al. undertook a study of 161 elderly patients with and without SZ. Beta diversity could be used to cluster the two groups distinctly and identify SZ-associated bacteria, including Faecalibacterium, Roseburia, Actinomyces, Butyricicoccus, and Prevotella. IL-1β, a proinflammatory cytokine, was greatly increased in SZ patients whereas anti-inflammatory cytokines such as IFN-γ were markedly decreased. Functional prediction indicated that the metabolic changes could lead to the production of immunomodulatory metabolites by the SZ microbiome. This study provided novel insights into structural and functional changes in SZ patients, paving the way for non-invasive diagnosis and the potential to use microbiome engineering as a target for treatment.

Several clinical and preclinical studies relevant to ASD have described alterations in fecal microbial populations; however, it has been unclear whether similarities occur across models and in humans with ASD and mouse models. Alamoudi et al. aimed to understand the similarities and differences between microbial profiles in humans and preclinical models of autism. A systematic review of the literature was undertaken to clarify the overlap or differences between previous clinical studies of the microbiome in patients with autism. Next, the fecal microbial communities were examined in a variety of mouse models of autism, including transgenic (i.e., NL3^R451C^, Shank3 KO, 15q dup mice), the behaviourally identified BTBR mouse model, and the Poly-I:C and Maternal Inflammation Activation (MIA) and valproate environmental models. These authors identified that some genera were altered in fecal samples from both human autism and mouse model studies.

Interestingly, *Bilophila, Clostridium, Dorea*, and *Lactobacillus* were increased in abundance in both clinical and mouse studies, whereas a decrease in *Blautia* was identified from the literature in both species. Some bacterial genera appeared to be susceptible to modification in both ASD patients and mouse models of autism, but the directional changes were non-uniform. These genera susceptible to change included *Akkermansia, Bacteroides, Bifidobacterium, Parabacteroides, and Prevotella*. Characterizing these changes in microbial profiles in both clinical cohorts and mice relevant to ASD can assist in designing potential therapies that modulate the gut microbial populations for individuals with ASD.

In a similar vein to Alamoudi et al. and Hashim and Makpol compared clinical and preclinical studies of the microbiome but in the context of neurodegenerative diseases and the current state of the field in terms of a range of approaches being explored to shape the gut microbiome. Potential therapeutic approaches examined in this review include the use of probiotics, dietary modifications, fecal microbiota transplantation (FMT), and alterations in physical activity with the view to reduce or prevent the effects of aging and neurodegenerative diseases. Specifically, microbial dysbiosis reported in clinical studies focusing on Alzheimer's Disease, Parkinson's Disease, ALS, and FTD were reviewed. The potential benefits of this selected group of interventions for reducing or preventing the progression of these diseases were discussed, given that significant correlative evidence supporting the role of the brain-gut microbiota (BGM) axis in these disorders exists.

In summary, these studies add to the growing evidence of a significant role for the microbiota-gut-brain axis in human health and highlight the critical need for multidisciplinary projects in this field.

## Author contributions

All authors listed have made a substantial, direct, and intellectual contribution to the work and approved it for publication.

